# Specific ion effects on the soil shear strength and clay surface properties of collapsing wall in Benggang

**DOI:** 10.7717/peerj.17796

**Published:** 2024-09-03

**Authors:** Bifei Huang, Maojin Yang, Honglin Zhong, Jinshi Lin, Fangshi Jiang, Ming-kuang Wang, Yue Zhang, Yanhe Huang

**Affiliations:** 1 Department of Forensic Science, Fujian Police College, Fuzhou, Fujian, China; 2 Jinshan Soil and Water Conservation Research Center, Fujian Agriculture and Forestry University, Fuzhou, Fujian, China

**Keywords:** Clay surface properties, Cohesion, Monovalent cation, Shear plane thickness, Stress–strain

## Abstract

Benggangs are a special type of soil erosion in the hilly granite regions of the tropical and subtropical areas of Southern China. They cause severe soil and water loss, which can severely deteriorate soil quality and threat to the local ecological environment. Soils (red soil, sandy soil and detritus soil) were collected from collapsing wall of a typical Benggang in Changting County of Fujian Province, and their physicochemical and mineralogical properties were analyzed. Five different monovalent cations were used to saturate the soil samples to examine the specific ion effects on the shear strength and clay surface properties. Red soil had a higher clay content, plastic limit, liquid limit and shear strength than sandy soil and detritus soil. The studied soils mainly consisted of kaolinite, hydroxy-interlayer vermiculite, illite and gibbsite clay minerals. The soils saturated with K^+^, NH_4_^+^and Cs^+^ had greater cohesion than the Li^+^- and Na^+^-saturated soils, *e.g.*, the cohesion of the red soil saturated with Li^+^, K^+^, NH_4_^+^ and Cs^+^ cations were 1.05, 1.23, 1.45 and 1.20 times larger than that of the Na^+^-saturated soil, respectively. While the internal friction angle was slightly different, which indicated that different monovalent cations affected the shear strength differently. K^+^-, NH_4_^+^- and Cs^+^-saturated clay particles had higher zeta potentials and thinner shear plane thicknesses than Li^+^- and Na^+^-saturated clay particles and showed strong specific ion effects on the clay surface properties. The changes in clay surface properties strongly affected the soil mechanical properties. Soils saturated with K^+^, NH_4_^+^ and Cs^+^ could increase the shear strength, and then increase the stability of the collapsing wall, thus might decrease the erosion intensity of Benggang. The results provide a scientific basis for the interpretation of and practical treatment of Benggang.

## Introduction

**Figure 1:**
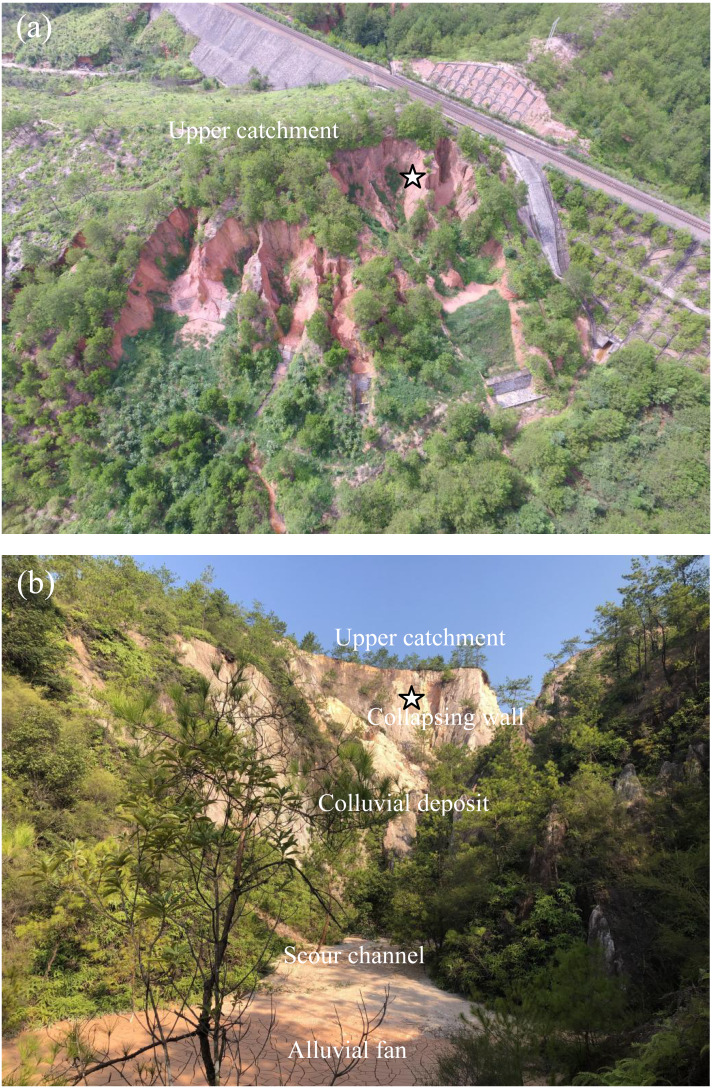
A typical Benggang in Fujian Province. (A) Aerial photograph of a Benggang in the study area; (B) Upper catchment, collapsing wall, colluvial deposit, scour channel and alluvial fan (Note: The five-pointed star represents the sampling location) Photo credit: Jinshi Lin.

Benggang erosion is one of the most remarkable and heavy erosion in the hilly granitic region of the tropical and subtropical areas of southern China, vividly described as an “ecological ulcer” (Wang et al., 2020; Zhang et al., 2020; Zhu et al., 2023). First described by Zeng in 1960, Benggang erosion is a type of composite erosion originally resulting from hydraulic scour and gravitational collapse (Zeng, 1960). A typical Benggang is composed of five parts: an upper catchment, a collapsing wall, a colluvial deposit, a scour channel and an alluvial fan (Fig. 1) (Xu, 1996; Sheng & Liao, 1997). It has been reported that there are approximately 239,100 permanent Benggangs throughout the hilly granitic region in various provinces of Southern China (Jiang et al., 2014). The annual erosion in these areas reaches approximately 50 kt km^−2^, which is over 50-fold higher than the erosion on gentler slopes or slopes with dense vegetation (Zhong et al., 2013). Moreover, from 1950 to 2005, Benggang erosion affected 1,220 km^2^ in the red granitic soil region and caused soil losses of more than 60 Mt. Benggang erosion is characterized by rapid development and sudden eruptions, and sometimes a rainstorm can nearly double the erosion area; therefore, it poses a greater threat than general soil erosion, leading to destruction of land resources (Liang et al., 2009). Obviously, Benggang erosion has caused great damage to the ecological environment and social development in the granitic red soil regions of South China.

Researchers who have carried out studies of Benggang have primarily focused on the damage and failure mechanism and found that Benggang erosion is closely related to the stability of collapsing walls; slumps and massive collapses of collapsing walls are among the main influential factors responsible for the amount of erosion and the development speed (Luk, Dicenzo & Liu, 1997a; Luk et al., 1997b; Sheng & Liao, 1997; Lan et al., 2003; Xia et al., 2015; Wei et al., 2021). Benggangs have a close relationship with the mechanical properties of soil, and shear strength is a primary factor controlling the stability of collapsing walls (Zhang, Ding & Cai, 2012; Deng et al., 2018; Huang et al., 2019; Zhang et al., 2020; Huang et al., 2022). Shear strength is a direct indicator of how shear deformation occurs under the action of external shear forces. When the shear stress reaches the shear strength, it will lead to the instability of the collapsing wall and cause collapse erosion (Deng et al., 2020). Studies have found that water content can seriously affect the shear strength and was closely related to the formation of Benggang (Dong et al., 2011; Chen et al., 2018; Gong et al., 2022).

**Figure 2:**
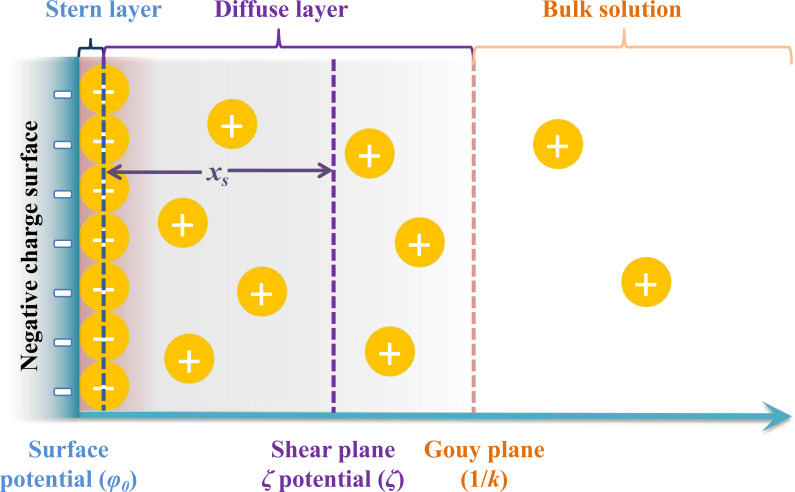
The schematic representation of the relative positions of zeta potential (*ζ*) and shear plane thickness (*x*_*s*_) in the double electric layer.

Moreover, a change in the water content of soils leads to a change in the ion concentration in the soil through chemical interactions between water and soil, which results in the redistribution of ions in these two matrices, leading to changes in the chemical properties and microstructure of surface soil particles and affecting soil mechanical properties (Mitchell & Soga, 1976; Gast, 1977; Wang et al., 2010; Wen & He, 2012; Yuan et al., 2022). Charged clay particles are the main solid components of soils. When charged clay particles disperse in an aqueous solution, they can absorb cations from the bulk solution and then form an electric double layer (EDL) around the charged clay particle surface (Van Olphen, 1977; Xu, 1998; Li et al., 2004). According to the theory of the electric double layer model, a shear plane exists between the electric double layer and the particle surface, and the potential at the shear plane is regarded as the zeta potential (*ζ*), while the distance from the shear plane to the particle surface is considered as the shear plane thickness (*x*_*s*_) (Fig. 2) (Sparnaay, 1972). The existence of a shear layer has a substantial effect on the stability of colloidal system soils, which would also affect the mechanical properties of soils. A large number of ions adsorbed on charged clay particles means that the surface reactions of ions profoundly affect the soil properties and interactions between clay particles. Marchuk & Rengasamy (2011) investigated the influence of different adsorbed cations on clay aggregates. For Li^+^-, Na^+^-, and K^+^-saturated clay aggregates, their turbidity, zeta potential and mean particle size after dispersion in water were highly related, following the order of Li^+^>Na^+^>K^+^, which led to different cation-exchange properties of the clays. Therefore, clay properties are closely related to water chemistry and surface charges. Different cation surface reactions at the ion-clay interfaces cause different effects on the surface properties, such as the zeta potential (*ζ*) and the shear plane thickness (*x*_*s*_) in the EDL of charged clay particles, and then show specific ion effects. Studies have reported that specific ion effects are significantly correlated with ionic properties, including ionic size and ionic polarization (Parsons et al., 2011; Rios-Carvajal et al., 2018). Nonclassically polarized adsorbed cations could increase the Coulomb interaction forces between cations and clay surfaces and reduce the electrostatic field strength surrounding the clay particles and the electrostatic repulsion between adjacent clay particles in aggregates, thus strongly increasing the aggregate stability of soils (Hu et al., 2015a; Li et al., 2015; Xu, Yu & Li, 2015a; Li, Li & Yang, 2017).

Different ion-clay interactions can cause different effects on the clay surface properties, thus strongly influencing the structures and stability of clay aggregates and mechanical properties. However, the specific ion effects on the shear strength and clay surface properties of Benggang remain obscure, which will lead to poorly understood of study the failure mechanism of Bengang and the process of soil and ecological environment restoration in Bengang area. Therefore, the objectives of this study were as follows: (1) investigate the physicochemical and mineralogical properties of the soil samples; (2) evaluate the specific ion effects of Li^+^, Na^+^, K^+^, NH_4_^+^ and Cs^+^ on the soil shear strength (*i.e.,* stress–strain characteristics, cohesion and internal friction angle); and (3) study the specific ion effects on clay surface properties, such as zeta potential (ζ) and shear plane thickness (*x*_*s*_). This study will help to understand the mechanism of soil mass failure of sidewalls in Benggang and provide practical treatments for Benggang erosion.

## Materials and methods

### Study area descriptions

**Figure 3:**
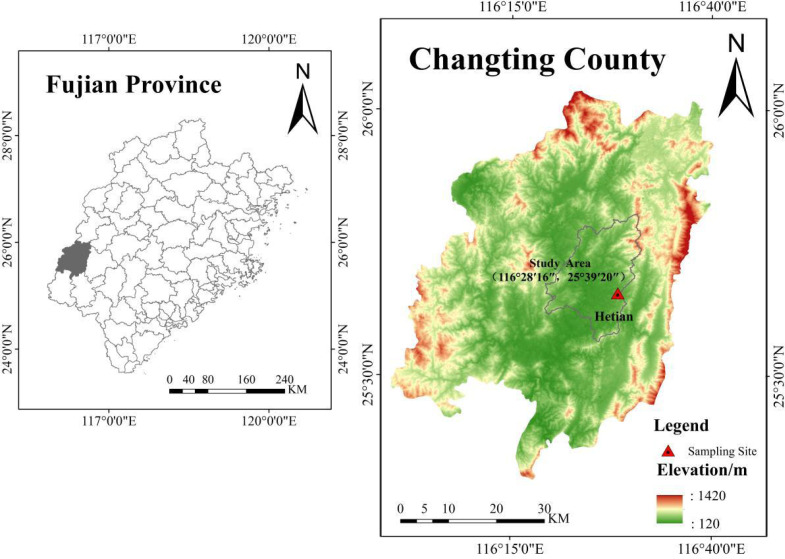
Location of Changting County of Fujian Province and Hetian town soil sampling site. Map data source credit: https://www.gscloud.cn/phonehome. Map created using ArcGis Software.

Experiments were conducted in Hetian town in Changting County (25°39′N, 116°28′E) in southwestern Fujian Province (Fig. 3). The area has a typical subtropical monsoon humid climate with an 18.3 °C mean annual temperature and a 1,730 mm average annual precipitation, and the rainfall is mainly concentrated from April to September. The soil in the study area is belong to the Ultisols, which is developing from acidic coarse-grained biotitic granite, and composes of feldspar, quartz and mica minerals; this type of soil accelerates weathering processes and slope failure when the protective vegetation cover is removed and triggers Benggang erosion.

### Soil samplings and analysis

A typical Benggang (25°39′20 ″N, 116°28′16″E) that was actively experiencing collapsing wall sliding was selected from Hetian town, Changting County (Fig. 3). The Benggang has an altitude of 314–320 m and a 55% vegetation coverage which is dominated by *Pennisetum sinese*, *Pinus massoniana* and *Dicranopteris linearis*. According to the color and structural characteristics of soil, the typical granitic profile of Benggang can be subdivided into three soil layers: red soil layer, sandy soil layer and detritus layer with increasing soil depth. Samples of the red soil, sandy soil and detritus soil were collected from the corresponding layers and brought back to the laboratory for physicochemical property analyses and pretreatments.

The soil samples were air-dried naturally and then ground to pass through a 2 mm sieve. The pH value of the soil samples was determined by a STARTER 2100 pH meter (OHAUS Instruments Co., Ltd., Shanghai, China) with 1:2.5 soil to water suspensions. Cation-exchange capacity (CEC) was determined after extraction with ammonium acetate (Rhoades, 1982). Soil organic matter (SOM) was measured by the K_2_Cr_2_O_7_-H_2_SO_4_ oxidation method (Jackson, 1979). The bulk density (BD) was determined by a metal cylinder with a 100 cm^3^ volume (diameter of 5.02 cm and height of 5.05 mm). The particle size distribution was measured by a BT-9300ST laser particle size analyzer (Bettersize Instruments Ltd., Liaoning, China). Plastic limits and liquid limits were determined by using a liquid/plastic limit tester. All tests were conducted in triplicate.

### X-ray diffraction analysis

The clay particles were collected by a gravity settling method according to Stokes’ Law (Jackson, 1979). Organic matter and Fe oxides were removed by 30% H_2_O_2_ and dithionite–citrate–bicarbonate (DCB) methods, respectively. The collected clay particles were centrifuged, washed with deionized water three times and then freeze dried.

X-ray diffraction (XRD) studies were carried out using an X-ray diffractometer (Ultima IV, Rigaku Corporation, Tokyo, Japan). The oriented clay samples were scanned from 3° to 40°2θ with a 1°2θ min^−1^ scan speed by using Cu-K α radiation generated at 40 kV and 40 mA. The slides of Mg-saturated clays were X-rayed at 25°C and further treated with glycerol solvation to identify expandable clay minerals. X-ray examination of the K-saturated clays was carried out at 25 °C and heated to 110, 250, 350, 450 and 550 °C for 2 h to characterize the kaolin minerals (Jackson, 1979; Pai et al., 1999). The quantity of vermiculite was determined by the CEC values of clays (Alexiades & Jackson, 1965). The K_2_O concentration was used to estimate the quantity of illite (Pai et al., 1999). The quantity of kaolinite was estimated by the XRD peak intensity at 7.1 Å (Brindley, 1980).

### Pretreatment of soil samples

Li^+^ (LiCl), Na^+^ (NaCl), K^+^ (KCl), NH_4_^+^ (NH_4_Cl) and Cs^+^ (CsCl) (analytical grade) were employed to investigate the specific ion effects on the shear strength and clay surface properties of the studied soils. It should be mentioned that Li^+^, Na^+^, K^+^, NH_4_^+^ and Cs^+^ were regarded as the best choice to investigate the specific ion effects of soils according to Hu et al. (2015b). The reasons are as follow: (i) the five cation species are monovalent and the Coulomb interactions are same, (ii) the size of Li^+^, Na^+^, K^+^, NH_4_^+^ and Cs^+^ cation species is different, indicates the ionic hydration effect is different, and (iii) NH_4_^+^ has similar ionic radii with K^+^, which can be used to investigate their effect on the shear strength and clay surface properties of Benggang soils. To prevent the influence of different anions, chloride salts of all cations were used to study the cation-specific behaviors.

In order to evaluate the specific ion effects on the shear strength and clay surface properties of Benggang soils. The LiCl, NaCl, KCl, NH_4_Cl and CsCl solution with a concentration of 0.1 mol L^−1^ were used to saturate the soil samples to obtain the Li^+^-, Na^+^-, K^+^-^−^, NH_4_^+^- and Cs^+^-saturated samples, respectively. It should be mentioned that solution with a concentration of 0.1 mol L^−1^ could make sure that the corresponding cation could absorb on the soil particle surface completely, which meant saturated treatment (Wu et al., 2018). The detail procedure was as follow: 3 kg of air-dried soil was weighed into a beaker, and 6 L of the corresponding cation solution was added. Then the solution was stirred for dispersion for 24 h and centrifuged (4,500 r min^−1^, 5 min) with the supernatant discarded. Then, this process was repeated three times to guarantee that the soil was saturated with monovalent cations, and residual metal cations were removed by dispersion, centrifugation and decantation with ultrapure water three times (Hu et al., 2015b). Thereafter, the saturated soil sample was dried in an oven at 60 °C for 48 h. The saturated soil samples were crushed to pass through a 2 mm sieve for the next experiments.

### Triaxial shear tests

Triaxial shear strength tests were performed with a TKA-TTS-3N triaxial compression system (Nanjing TKA Technology Co., Ltd., Nanjing, China) based on the unconsolidated-undrained (UU) test (Wei et al., 2018). The sample water content was controlled at 15% by weight by a field survey, and the corresponding soil density was determined based on the natural bulk density. The soil samples were divided into four portions and repacked into a cylinder-shaped sampler (diameter of 39.1 mm, height of 80 mm, cross-sectional area of 12 cm^2^). After each compaction, the surface of the soil samples was shaved to enhance the frictional bite between the samples. Shear strength triaxial tests were conducted under four confining stresses (50, 100, 200 and 300 kPa) with a shear velocity of 0.4 mm min^−1^. The experiments were conducted in triplicate. The data obtained from shear strength triaxial tests of samples were drawn into a circle under the same coordinate system, and then the common tangent line of circle could be drawn (Fredlund & Rahardjo, 1993). Therefore, the soil cohesion and internal friction angle could be observed by the intercept and slope.

### Sodium/calcium exchange equilibrium to determine the surface properties of clay particles

The freeze-dried clay particles were exchanged with the concentration of 0.1 mol L^−1^ HCl solution three times to obtain the H-saturated sample. A given quantity of the H-saturated sample was placed into an Erlenmeyer flask to perform the cation exchange experiment. Then, 15 mL of 0.02 mol L^−1^ Ca(OH)_2_ solution was added into the flask, and the suspensions were equilibrated for 12 h under continuous shaking to convert the H-saturated sample into a Ca-saturated sample. Then, 15 mL 0.02 mol L^−1^ NaOH solution was added and oscillated for 24 h. HCl solution was used to adjust the pH of suspension to 6.0∼8.0 while the pH was higher than 7 in equilibrium state. To obtain the exchange equilibrium state, the suspension was continually shaken for 12 h. The supernatant was collected by centrifugation (4500 r min^−1^, 5 min), and the Na^+^ and Ca^2+^ concentrations in the supernatant were measured by inductively coupled plasma optical emission spectrometry (ICP −OES) (Optima 8000, Perkin Elmer, New York). Therefore, the number of Na^+^ (*N*_*Na*_) and Ca^2+^ (*N*_*Ca*_) adsorbed on the charged clay surface could be obtained (Li et al., 2011). The experiments were conducted in triplicate.

### Theories and calculations of the surface properties of clay particles

The surface properties of clay particles can be calculated according to a combined measurement based on the sodium/calcium exchange equilibrium as follows (Li et al., 2011).

(1) Surface potential (*φ*_0_) (mV)

(1) \begin{eqnarray*}{\varphi }_{0}= \frac{2RT}{ \left( 2{\beta }_{Ca}-{\beta }_{Na} \right) F} \ln \nolimits \frac{{c}_{Ca}^{0}{N}_{Na}}{{c}_{Na}^{0}{N}_{Ca}} \end{eqnarray*}

where ${c}_{Na}^{0}$ and ${c}_{Ca}^{0}$ are the concentrations of the Na^+^ and Ca^2+^ in bulk solution (mol L^−1^), respectively; *R* is the molar gas constant (*R*= 8.314 J K^−1^ mol^−1^); *T* is the absolute temperature (K); *F* is the Faraday constant (*F*= 96485 C mol^−1^); *N*_*Na*_ and *N*_*Ca*_ are the numbers of Na^+^ and Ca^2+^ adsorbed on the particle surface, respectively; *β*_*Na*_ and *β*_*Ca*_ are the modification factors *β*_*Na*_ = 2 − *β*_*Ca*_ and *β*_*Ca*_ = 0.0213ln*I*^1/2^ + 1.2331, respectively.

(2) Specific surface area (*S*) (m^2^kg^−1^)

(2) \begin{eqnarray*}S= \frac{{N}_{Na}\kappa }{m{c}_{Ca}^{0}} e \frac{{\beta }_{Na}F{\varphi }_{0}}{2RT} \end{eqnarray*}

where *κ* is the Debye-Huckel parameter (dm^−1^), *κ* is defined as:

\begin{eqnarray*}\kappa =\sqrt{ \frac{8\pi {F}^{2}I}{RT} } \end{eqnarray*}

where *I* is the ionic strength; *ɛ* is the dielectric constant of water (8.9 × 10^−10^C^2^ J^−1^ dm^−1^); and *m* is the modification factor $m=0.5259\ln ({c}_{Na}^{0}/{c}_{Ca}^{0})+1.992$.

(3) Surface charge number (*SCN*) (cmol kg^−1^)

(3) \begin{eqnarray*}SCN={N}_{Na}+2{N}_{Ca}\end{eqnarray*}

(4) Surface charge density (*σ*_0_) (C m^−2^)

(4) \begin{eqnarray*}{\sigma }_{0}= \frac{SCN\times F\times 1{0}^{-5}}{S} \end{eqnarray*}

(5) Electrostatic field strength (*E*_0_) (V m ^−1^)

(5) \begin{eqnarray*}{E}_{0}= \frac{4\pi }{} {\sigma }_{0}.\end{eqnarray*}

The zeta potential (*ζ*) of clay particles is determined by a Nanobrook Omni zeta potential analyzer (Brookhaven Instruments Corporation, New York) in 0.0002, 0.0005, 0.001, 0.005 and 0.01 mol L^−1^ NaNO_3_ solutions. The experiments were conducted in triplicate. Then the shear plane thickness (*x*_*s*_) in different NaNO_3_ electrolyte solutions can be calculated by the follow equations (Li et al., 2011).

(6) \begin{eqnarray*}{x}_{s}=- \frac{1}{\kappa } \ln \nolimits [ \frac{1-{e}^{\zeta F/2RT}}{(1+{e}^{\zeta F/2RT}){\lambda }_{1:1}} ]\end{eqnarray*}

where *ζ* is the zeta potential (mV) and *λ*_1:1_ is a constant, which can be expressed as:

(7) \begin{eqnarray*}{\lambda }_{1:1}= \frac{1-{e}^{{\varphi }_{0}F/2RT}}{1+{e}^{{\varphi }_{0}F/2RT}} \end{eqnarray*}

where *φ*_0_is the surface potential (mV) in different NaNO_3_ electrolyte solutions, which can be expressed as:

(8) \begin{eqnarray*}{\varphi }_{0}= \frac{2RT}{F} \ln \nolimits \frac{2{c}^{0}}{{\sigma }_{0}\kappa } \end{eqnarray*}

where *c*^0^ is the concentration (mol L^−1^) of the different NaNO_3_ electrolyte solutions.

### Statistical analyses

Origin 8.5 software was used to draw figures. The SPSS 18.0 software package was used to perform the data analysis, and one-way ANOVA was performed at a significance level of *P* < 0.05 to determine if significant differences existed among different treatments.

## Results

### Soil physicochemical and mineralogical properties

#### Soil physicochemical properties

Soil physical and chemical properties are summarized in Table 1. The pH values of red soil, sandy soil and detritus soil were 5.06, 5.13 and 5.25, respectively. The SOM and CEC in the red soil (3.28 g kg^−1^ and 10.94 cmol kg^−1^) were higher than those in the sandy soil (1.10 g kg^−1^and 7.25 cmol kg^−1^) and detritus soil (1.21 g kg^−1^ and 6.90 cmol kg^−1^). The BD of the detritus soil (1.43 g cm^−3^) was higher than that of the sandy soil (1.32 g cm^−3^) and red soil (1.32 g cm^−3^). The red soil had a higher clay content (26.93%) and lower sand content (20.40%) than the sandy soil (7.32%, 40.62%) and detritus soil (7.48%, 38.35%). The plastic limit of the red soil, sandy soil and detritus soil was 39.32%, 29.55% and 29.16%, the liquid limit of the red soil, sandy soil and detritus soil was 84.27%, 47.90% and 40.11%, and the plasticity index of the red soil, sandy soil and detritus soil was 44.95%, 18.35% and 10.95%, respectively. These results were similar to those reported by Xia et al. (2019).

**Table 1:** Physical and chemical properties of the studied soils.

Soil layer	pH	SOM^1^ g kg^−1^	CEC cmol kg^−1^	BDg cm^−3^	Sand	Silt	Clay	Texture	PL	LL	PI
					———————-%————————–				———————%———————–		
Red soil	5.06 ± 0.08	3.28 ± 0.01	10.94 ± 0.11	1.32 ± 0.01	20.40 ± 0.45	52.67 ± 0.76	26.93 ± 0.77	SL	39.32 ± 0.49	84.27 ± 0.13	44.95 ± 0.57
Sandy soil	5.13 ± 0.07	1.10 ± 0.03	7.25 ± 0.19	1.32 ± 0.02	40.62 ± 0.27	52.06 ± 0.23	7.32 ± 0.05	SL	29.55 ± 0.17	47.90 ± 0.07	18.35 ± 0.23
Detritus soil	5.25 ± 0.04	1.21 ± 0.01	6.90 ± 0.05	1.43 ± 0.01	38.35 ± 0.66	54.18 ± 0.58	7.48 ± 0.09	SL	29.16 ± 0.15	40.11 ± 0.15	10.95 ± 0.26

**Notes.**

1 SOM, soil organic matter; CEC, cation-exchange capacity; BD, bulk density; SL, silty loam; PL, plastic limit; LL, liquid limit; PI, plasticity index.

#### Identification of mineralogical properties by XRD

**Figure 4:**
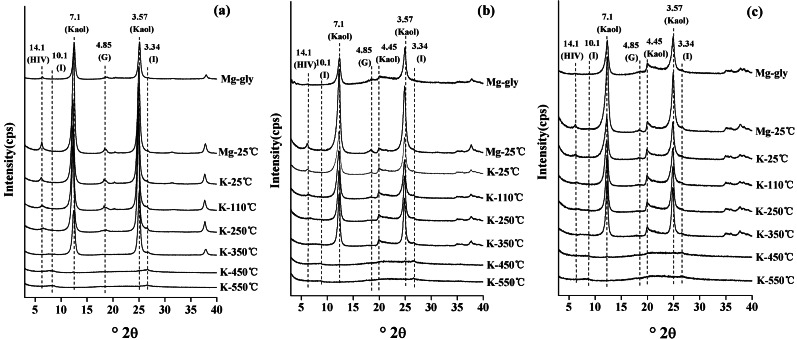
The X-ray oriented diffraction patterns of clay particles of red soil (A), sandy soil (B) and detritus soil (C). HIV, hydroxy-interlayered vermiculite; I, illite; Kaol, kaolinite; G, gibbsite.

XRD diffraction patterns of three clay samples (red soil, sandy soil and detritus soil) are shown in Fig. 4. The 001 reflection of 14.1 Å at 25 °C of the Mg-saturated clay samples with glycerol solvation did not shift to a higher *d*-value, which indicated that there were no expandable clay minerals in the clay samples. The XRD patterns indicated the presence of hydroxy-interlayer vermiculite (HIV), which was characterized by the XRD reflection of 14.1 Å at 25 °C, collapsing to 10.1 Å completely when the K-saturated clays were heated to 350 °C. The 001 reflection of illite was at 10.1 Å in the K-saturated clay sample after heating at 110 and 550 °C. The 001 reflection at 7.1 Å was not observed after heating the K-saturated clays at 550 °C, indicating that kaolinite was present in the clay samples. The XRD reflection of 4.85 Å was not observed after heating to 350 °C, which indicated the presence of gibbsite. Therefore, the results showed that the studied soil mainly consisted of four kinds of clay minerals: hydroxy-interlayer vermiculite, illite, kaolinite and gibbsite. In addition, the semiquantitative analysis of the mineralogical compositions of clay samples is shown in Table 2. The amount of kaolinite in red soil (86.19%) was higher than that in sandy soil (81.82%) and detritus soil (82.32%).

**Table 2:** Semiquantitative analysis of clay minerals in the soil clay fractions of the studied soils by XRD patterns using oriented specimens.

Soil layer	Clay mineral (%)			
	Kaolinite	HIV^*^	Illite	Gibbsite
Red soil	86.19	6.63	2.76	4.42
Sandy soil	81.82	8.02	4.81	5.35
Detritus soil	82.32	8.08	5.05	4.55

**Notes.**

* HIV, hydroxy-interlayer vermiculite.

### Effects of the different monovalent cations on shear strength

#### Effects of the different monovalent cations on soil stress–strain characteristics

**Figure 5:**
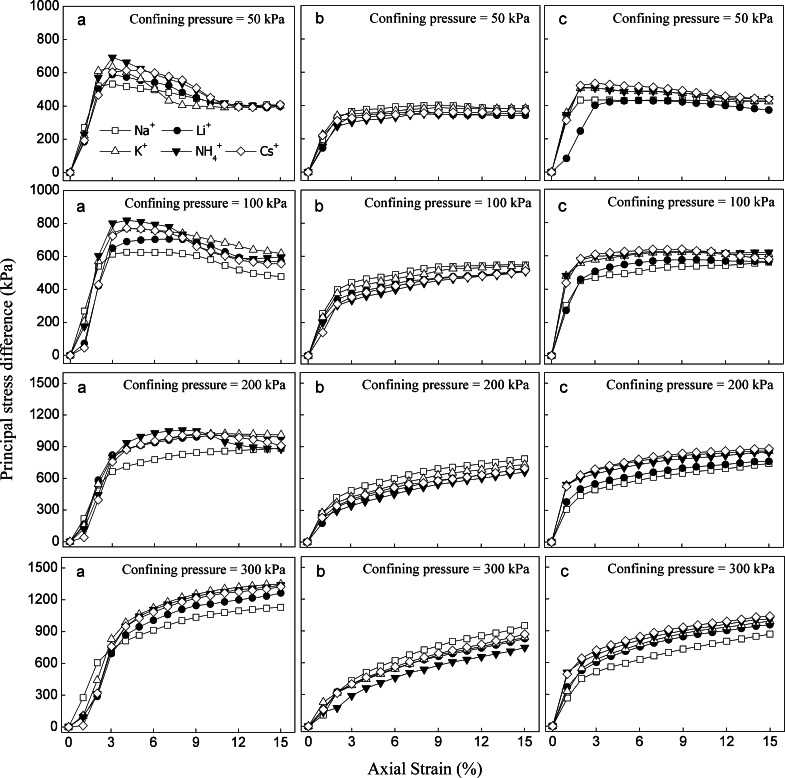
Stress-strain curves of the studied soils saturated with different monovalent cations. (A) Red soil; (B) Sandy soil; (C) Detritus soil.

The stress–strain curve was used as an indicator to reflect the shear strength and failure type of the soil samples during triaxial shear testing. While the principal stress difference increases up to a maximum and then decreases with an increase in strain, the type of stress–strain curve shows the softening strain. However, the principal stress difference increases continuously with increasing strain until failure, and the stress–strain morphology hardens under this condition. Figure 5 shows the stress–strain curves for soils saturated with monovalent cations. The stress–strain curve of soil was significantly affected by different monovalent cations. For red soils (Fig. 5A), there were obvious peaks in the stress–strain curves of soils saturated with five different monovalent cations and displayed softening stress–strain curves when confining pressures were lower (50 and 100 kPa). The stress–strain curve exhibited strain-softening characteristics except for the Na^+^-saturated soil when the confining pressure was 200 kPa. However, the hardening stress–strain curve was observed when the confining pressure was 300 kPa. At the same confining pressure, the principal stress difference decreased in the following order: NH_4_^+^>Cs^+^>K^+^>Li^+^>Na^+^ in red soils. The situation was quite different in sandy soils saturated with different monovalent cations (Fig. 5B). The stress–strain curve showed strain–hardening under all four confining pressures (50, 100, 200 and 300 kPa), and the principal stress difference decreased as follow: Na^+^ >K^+^>Cs^+^>Li^+^ >NH_4_^+^ at the same confining pressure. In detritus soils (Fig. 5C), softening stress–strain curves were obtained for soils saturated with monovalent cations, and the hardening curve occurred in Na^+^-saturated soil only when the confining pressure was 50 and 100 kPa, whereas when the confining pressure was 200 and 300 kPa, the stress–strain curves showed strain–hardening of the detritus soils saturated with different monovalent cations. The principal stress difference decreased following the order of Cs^+^ >NH_4_^+^ >K^+^ >Li^+^ >Na^+^ in detritus soils with the same confining pressure.

#### Effects of the different monovalent cations on cohesion and internal friction angle

**Figure 6:**
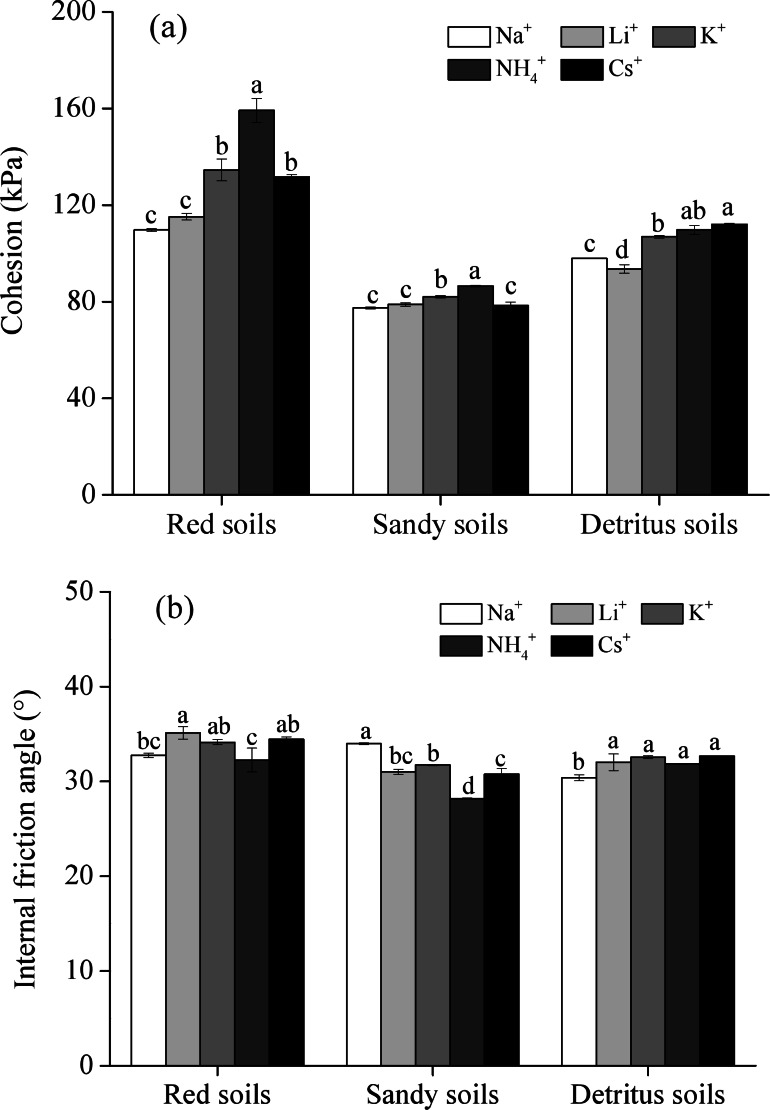
The shear strength of soils saturated with different monovalent cations. (A) cohesion; (B) internal friction angle) (Note: different letters represent significant differences among different cations of the same soil layers (*P* < 0.05)).

The shear strength (cohesion and internal friction angle) of the soil saturated with five different monovalent cations is shown in Fig. 6. The cohesion (*c*) of red soils ranged from 109.66 to 159.17 kPa and that of sandy soils and detritus soils ranged from 77.43 to 86.48 kPa and 93.57 to 112.05 kPa, indicating that the cohesion of red soils was much higher than that of sandy soils and detritus soils. This was attributed to the SOM and clay contents decreasing as the soil depth increasing (Rahardjo et al., 2012; Yu & Xu, 2016). The internal friction angle (*φ*) of red soils (32.26° to 35.11°) was greater than those in sandy soils (28.19° to 33.98°) and detritus soils (30.38° to 32.65°). Compared with sandy soil layer and detritus layer, red soil layer had the higher shear strength, indicating that the red soil layer had a higher resistance to Banggang erosion from external forces among the three soil layers. Therefore, once the red soil layer (upper soil layer) was washed away, the gully head and sidewall were prone to sliding because of the low shear strength of the sandy soil layer and detritus layer (lower soil layers), especially under water-saturated conditions (Xia et al., 2019). Moreover, under rainfall conditions, the sandy soil layer and detritus layer were deposited prior to erosion, scouring the lower soil layers and suspending the upper red soil layer. When the weight of suspended red soils exceeded the shear strength, collapse erosion occurred (Chen et al., 2015).

Although Li^+^, Na^+^, K^+^, NH_4_^+^ and Cs^+^ are all monovalent cations, the influences of the five different monovalent cations on the shear strength are different. For example, the cohesion (*c*) of the red soil saturated with Na^+^, Li^+^, K^+^, NH_4_^+^, and Cs^+^ monovalent cations was 109.66, 115.20, 134.56, 159.17 and 131.64 kPa, respectively, which meant that the *c* values of red soil saturated with Li^+^, K^+^, NH_4_^+^, and Cs^+^ cations were 1.05, 1.23, 1.45 and 1.20 times larger, respectively, than that of the Na^+^ saturated soil. The *c* value increased with different monovalent cations in the following order: NH_4_^+^ >K^+^ >Cs^+^ >Li^+^ ≈ Na^+^. However, the internal friction angle (*φ*) showed the opposite trend in red soils and sandy soils, and there were no significant differences in *φ* values in detritus soils.

### Effects of the different monovalent cations on clay surface properties

The combined measurement method reported by Li et al. (2011) was employed to calculate the surface properties of clay particles. The calculated values for ionic strength (*I*), modification factor (*m*), concentration of bulk solution (${c}_{Na}^{0}$ and ${c}_{Ca}^{0}$) and adsorbed cations (*N*_*Na*_ and *N*_*Ca*_) of the diffuse layer at the clay surface under neutral solution are shown in Table 3. After introducing the corresponding data into Eqs. (1) to (5), the observed values for the surface properties (*i.e.,* surface potential (*φ*_0_), specific surface area (*S*), surface charge number (*SCN*), surface charge density (*σ*_0_) and electrostatic field strength (*E*_0_)) of three different soil layers are summarized in Table 4. The surface potential (*φ*_0_) was −89.74 mV in red soil, −84.96 mV in sandy soil and -85.93 mV in detritus soil. The specific surface areas (*S*) of red soil, sandy soil and detritus soil were 33.45 ×10^3^, 21.58 ×10^3^ and 21.20 × 10^3^ m^2^ kg^−1^, respectively. The surface charge number (*SCN*) of the red soil, sandy soil and detritus soil were 26.06, 24.37 and 22.92 cmol kg^−1^, respectively. The *σ*_0_ and *E*_0_ of red soil were 0.73 C m^−2^ and 9.24 × 10^8^ V m^−1^, respectively, while the *σ*_0_ and *E*_0_ of sandy soil were 1.12 C m^−2^ and 15.86 × 10^8^ V m^−1^, 1.05 C m^−2^ and 14.76 × 10^8^ V m^−1^ in detritus soil.

**Table 3:** Ion exchange equilibrium calculation results of clay particles for the studied soils under neutral conditions (*n* = 3).

Soil layer	*c*_*Na*_^0^	*c*_*Ca*_^0^	*N*_*Na*_	*N*_*Ca*_	^1^ *I*	*m*	1/ κ
	mmol L^−1^		10^−2^ mol kg^−1^		10^−3^		nm
Red soil	8.89 ± 0.06	2.26 ± 0.16	1.60 ± 0.09	11.59 ± 0.24	8.97 ± 0.34	2.71 ± 0.03	3.32 ± 0.07
Sandy soil	9.06 ± 0.16	3.26 ± 0.22	1.31 ± 0.25	10.08 ± 0.33	11.04 ± 0.51	2.53 ± 0.03	3.18 ± 0.06
Detritus soil	9.05 ± 0.21	2.78 ± 0.53	1.29 ± 0.36	10.78 ± 0.82	10.09 ± 1.17	2.62 ± 0.08	3.37 ± 0.03

**Notes.**

1 *I*, ionic strength; *m*, modification factor.

**Table 4:** Surface properties of clay particles for the studied soils under neutral conditions (*n* = 3).

Soil layer	*φ*_0_	*S*	*SCN*	*σ*_0_	*E*_0_
	mV	10^3^m^2^kg^−1^	cmol kg^−1^	C m^−2^	10^8^ V m^−1^
Red soil	−89.74 ± 4.74	33.45 ± 0.29	26.06 ± 1.20	0.73 ± 0.01	9.24 ± 1.90
Sandy soil	−84.96 ± 1.17	21.58 ± 5.23	24.37 ± 0.13	1.12 ± 0.28	15.86 ± 3.93
Detritus soil	−85.93 ± 1.86	21.20 ± 1.28	22.92 ± 0.34	1.05 ± 0.05	14.76 ± 0.66

**Notes.**

*φ*_0_ surface potential S surface area SCN surface charge number *σ*_0_ surface charge density *E*_0_ electrostatic field strength

The zeta potential (*ζ*) of the clay particles saturated with five different monovalent cations can be determined using a zeta potential analyzer with the different electrolyte concentration solutions. The results for the measured zeta potential are shown in Table 5. The zeta potential (*ζ*) of the clay particles ranged from −32.02 to −56.92 mV, and the result was in accordance with previous reports (Xu & Xiao, 2009), which showed that the zeta potential of charged clay particles ranged from −15 to −60 mV. The absolute zeta potential of the saturated clay particles decreased as the electrolyte concentration increased. The absolute zeta potential of the detritus soils was higher than that of the sandy soils and was lowest in the red soils. For instance, in 0.0002 mol L^−1^ NaNO_3_ solutions, the zeta potential ranged from −41.20 to −51.49 mV in red soils, −47.36 to −56.92 mV in sandy soils and −49.28 to −54.80 mV in detritus soils. Moreover, the absolute zeta potentials of clay particles in the studied soils saturated with Li^+^, Na^+^, K^+^, NH_4_^+^ and Cs^+^ were obviously different in solutions with the same electrolyte concentration, indicating strong specific ion effects on zeta potential, which showed the following order: Cs^+^ >K^+^ >NH_4_^+^ >Na^+^ >Li^+^.

**Table 5:** Zeta potential (*ζ*) of clay particles for the studied soils saturated with different monovalent cations in NaNO_3_ solution at different concentrations.

Soil layer	Saturated cation	0.0002 mol L^−1^	0.0005 mol L^−1^	0.001 mol L^−1^	0.005 mol L^−1^		0.01 mol L^−1^
		*ζ*(mV)	*ζ*(mV)	*ζ* (mV)		*ζ*(mV)		*ζ*(mV)
Red soil	Na^+^	−42.88 ± 1.91	−41.48 ± 1.92	−38.30 ± 0.66	−36.79 ± 1.37		−36.05 ± 0.57
	Li^+^	−41.20 ± 0.45	−41.82 ± 0.60	−35.58 ± 0.72	−35.95 ± 0.45		−36.43 ± 0.44
	K^+^	−45.71 ± 0.39	−42.75 ± 2.20	−43.41 ± 1.37	−37.62 ± 0.62		−32.02 ± 0.83
	NH_4_^+^	−47.91 ± 0.89	−46.11 ± 0.55	−42.11 ± 0.26	−37.82 ± 1.07		−36.74 ± 0.86
	Cs^+^	−51.49 ± 1.97	−48.50 ± 0.41	−46.06 ± 1.57	−42.09 ± 0.76		−38.98 ± 1.81
Sandy soil	Na^+^	−47.36 ± 1.42	−47.49 ± 1.84	−46.16 ± 0.73	−43.54 ± 2.07		−43.84 ± 1.86
	Li^+^	−54.19 ± 0.48	−52.42 ± 0.97	−51.82 ± 0.69	−47.77 ± 0.67		−45.69 ± 0.57
	K^+^	−56.92 ± 2.31	−54.64 ± 1.02	−53.82 ± 0.91	−52.67 ± 0.79		−49.37 ± 0.08
	NH_4_^+^	−54.03 ± 1.89	−49.80 ± 1.25	−49.81 ± 1.63	−49.55 ± 2.06		−44.55 ± 0.65
	Cs^+^	−52.06 ± 2.41	−50.41 ± 1.55	−45.85 ± 0.80	−43.47 ± 1.24		−42.52 ± 0.58
Detritus soil	Na^+^	−51.10 ± 0.51	−51.38 ± 0.99	−49.50 ± 0.69	−49.35 ± 0.71		−47.14 ± 1.48
	Li^+^	−49.28 ± 1.41	−51.89 ± 2.11	−53.04 ± 2.04	−47.81 ± 1.87		−44.72 ± 0.96
	K^+^	−54.67 ± 0.71	−50.26 ± 2.20	−51.71 ± 0.21	−44.86 ± 1.20		−44.87 ± 0.33
	NH_4_^+^	−54.12 ± 1.30	−50.68 ± 0.79	−50.76 ± 1.40	−44.77 ± 0.67		−43.59 ± 1.53
	Cs^+^	−54.80 ± 0.32	−51.42 ± 0.24	−50.38 ± 0.47	−47.20 ± 0.93		−46.35 ± 1.11

Then the obtained values of surface potential and zeta potential of clay particles were introduced into Eq. (6), the shear plane thickness (*x*_*s*_) in different electrolyte concentration solutions could be calculated and was shown in Table 6. With increasing electrolyte concentration, the shear layer of clay particles compressed (became thinner), which decreased the shear plane thickness of clay particles. For example, in soils saturated with K^+^, the *x*_*s*_ value for red soils ranged from 18.91 to 3.63 nm in 0.0002 to 0.01 mol L^−1^ NaNO_3_ solutions, ranged from 14.88 to 2.44 nm in sandy soils and ranged from 15.59 to 2.70 nm in detritus soils. For a solution with a given electrolyte concentration, the *x*_*s*_ values of clay particles of the studied soils saturated with Li^+^, Na^+^, K^+^, NH_4_^+^ and Cs^+^ were quite different. Intensifying specific ion effects on shear plane thickness were observed. The result showed that the shear plane thickness values of clay particles of studied soils saturated with Li^+^ and Na^+^ were higher than those saturated with K^+^, NH_4_^+^ and Cs^+^.

**Table 6:** Shear plane thickness (*x*_*s*_) of clay particles for the studied soils saturated with different monovalent cations in NaNO_3_ solution at different concentrations.

Soil layer	Saturated cation	0.0002 mol L^−1^	0.0005 mol L^−1^	0.001 mol L^−1^	0.005 mol L^−1^		0.01 mol L^−1^
		*x*_s_ (nm)	*x*_s_ (nm)	*x*_s_ (nm)		*x*_s_ (nm)		*x*_s_ (nm)
Red soil	Na^+^	20.15 ± 0.87	13.14 ± 0.57	9.97 ± 0.15	4.59 ± 0.15		3.29 ± 0.04
	Li^+^	20.92 ± 0.21	13.03 ± 0.18	10.63 ± 0.18	4.68 ± 0.05		3.26 ± 0.03
	K^+^	18.91 ± 0.16	12.77 ± 0.64	8.88 ± 0.27	4.50 ± 0.06		3.63 ± 0.07
	NH_4_^+^	18.02 ± 0.35	11.84 ± 0.14	9.14 ± 0.05	4.48 ± 0.11		3.24 ± 0.07
	Cs^+^	16.69 ± 0.71	11.23 ± 0.10	8.37 ± 0.29	4.06 ± 0.07		3.07 ± 0.13
Sandy soil	Na^+^	18.26 ± 0.56	11.51 ± 0.47	8.36 ± 0.13	3.95 ± 0.19		2.76 ± 0.12
	Li^+^	15.76 ± 0.16	10.34 ± 0.22	7.39 ± 0.11	3.59 ± 0.05		2.65 ± 0.03
	K^+^	14.88 ± 0.72	9.86 ± 0.21	7.08 ± 0.14	3.23 ± 0.05		2.44 ± 0.00
	NH_4_^+^	15.82 ± 0.64	10.94 ± 0.30	7.73 ± 0.27	3.46 ± 0.15		2.72 ± 0.04
	Cs^+^	15.51 ± 0.87	10.80 ± 0.36	7.42 ± 0.15	3.95 ± 0.11		2.85 ± 0.04
Detritus soil	Na^+^	16.83 ± 0.18	10.57 ± 0.22	7.77 ± 0.12	3.47 ± 0.05		2.57 ± 0.09
	Li^+^	17.42 ± 0.72	10.21 ± 0.30	7.24 ± 0.44	3.51 ± 0.09		2.68 ± 0.03
	K^+^	15.59 ± 0.24	10.84 ± 0.52	7.41 ± 0.03	3.83 ± 0.10		2.70 ± 0.02
	NH_4_^+^	15.78 ± 0.44	10.73 ± 0.18	7.57 ± 0.23	3.84 ± 0.06		2.78 ± 0.10
	Cs^+^	15.55 ± 0.11	10.56 ± 0.06	7.63 ± 0.08	3.64 ± 0.08		2.61 ± 0.07

## Discussion

### Vertical variations of granite residual soil properties of Benggang

The red soil has superior soil characteristics (*e.g.*, SOM, CEC and clay content) compared with sandy soil and detritus soil (Table 1). The red soil had the highest soil atterberg limit (plastic limit, liquid limit and the plasticity index), followed by the sandy soil and the detritus soil (Table 1), indicated that the soil atterberg limit of the soils were decreased noticeably as the soil depth increased, which could result from the relatively low SOM and clay contents in the sandy soil and detritus soil (Hemmat et al., 2010; Stanchi, Freppaz & Zanini, 2012). Deeper soil horizons had lower CEC, SOM, clay contents, plastic limits and liquid limits, indicating their poor soil structure, which is vulnerable to erosion under rainfall conditions. The XRD results showed that the clay mineral composition of soils mainly consisted of hydroxy-interlayer vermiculite, illite, kaolinite and gibbsite (Fig. 4). The kaolinite accounted for >81.82% of all clay minerals in the studied soils (Table 2), which meant that the Benggang soils were highly weathered. These results were in accordance with those reported by Chen et al. (2018) and Xia et al. (2019).

### Specific ion effects on the shear strength

**Figure 7:**
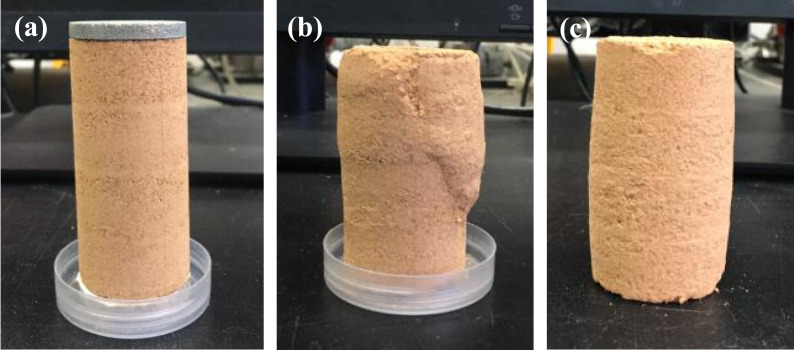
Specimens before and after triaxial shearing. (A) Before shearing; (B) Fracture breakage; (C) Plastic damage.

For the three soil layers, the principal stress difference of the red soil layer was greater than sandy soil layer and detritus layer at the same confining pressure, indicating a reduction of the shear strength of sandy and detritus soils. With the confining pressure increased, the stress–strain curves gradually changed from strain–softening to strain–hardening, strain-softening occurred less frequently, while the strain-hardening occurred more frequently, and the failure type of soil changed from fracture breakage to plastic damage (Fig. 7). In the triaxial shear tests, deformation of the soil specimen was more obvious under strain–softening and shear dilatancy with lower confining pressures. This result was consistent with that reported by Lai, Liao & Hu (2016) and Wei et al. (2018). As mentioned above, the principal stress difference of stress–strain curve is an indicator of shear strength. The stress–strain curve of the studied soils was significantly affected by different monovalent cations, indicated that cation became an important influence on soil shear strength. *e.g.*, the principal stress differences of red soils saturated with NH_4_^+^, Cs^+^, and K^+^ cation were higher then the Li^+^, Na^+^-saturated soils (Fig. 5A). The highly hydrated cations (Li^+^, Na^+^) adsorbed directly on the particle surface could form a thick but weak hydration layer, while poorly hydrated cations (NH_4_^+^, Cs^+^, K^+^) formed a thin but strong hydration layer, and the repulsive force increased with the thickness of hydration layer increased, which leading to the decrease of principal stress difference (Vakarelski, Ishimura & Higashitani, 2000). The principal stress difference of the three different soil layers showed different tendencies with cations because of the different physical properties and chemical properties three different different soil layers.

The shear strength (cohesion and internal friction angle) of the studied soils saturated with five electrolyte solutions was quite different, which meant that the different monovalent cations had different effects on the shear strength, indicating strong specific ion effects on the shear strength. The cohesion (*c*) value increased with different monovalent cations followed the sequence of NH_4_^+^ >K^+^ >Cs^+^ >Li^+^ ≈ Na^+^, while the internal friction angle (*φ*) showed the opposite trend in red soils and sandy soils, and there were no significant differences in *φ* values in detritus soils (Fig. 6), which indicated the soil shear strength has a close relationship with the cationic composition of soils. Therefore, these cations adsorbed on soil particles play a crucial role in Benggang erosion.

The previous studies had found that the cementing materials and electrostatic attraction between soil particles were seriously affected the shear strength (Tang et al., 2011; Zhang, Ding & Cai, 2012). An EDL formed surrounding the particles because of the adsorption of counterions from the bulk solution near the clay surfaces once the charged clay particles dispersed in an aqueous solution. The EDL overlaps when the clay particles are close to each other, and electrostatic repulsion between the clay particles can be generated to prevent the particles from approaching each other, which leads to an increase in clay particle dispersion and a decrease in the clay aggregate stability, thus decreasing the shear strength. The cation polarization could reduce the electric field strength around the surface of clay particles and the electrostatic repulsion between adjacent particles in the clay aggregates and increase the Coulomb adsorption forces between the clay particles and cations, thus increasing the stability of the clay aggregates (Hu et al., 2015b; Li et al., 2015; Xu et al., 2015b). Cations can be hydrated in an aqueous solution, which reduces their ability compared to ions that are not hydrated to shield the electric fields arising from charged clay particles. In addition, cations with a small hydrated radius are better able to shield the electric field than those with a large hydrated radius. Therefore, the electrostatic repulsion shows a positive relationship with the ionic hydrated radius. The size of these five cationic species decreases in the order of Cs^+^ (1.67 Å) >NH_4_^+^ (1.43 Å) >K^+^ (1.33 Å) >Na^+^ (0.97 Å) >Li^+^ (0.68 Å), indicating that the ionic hydration effects decreases in the following order: Li^+^ >Na^+^ >K^+^ >NH_4_^+^ >Cs^+^ (Bohn, McNeal & O’Connor, 2001). The ability of the five cations to shield the electric field strength and maintain the stability of clay aggregates decreases in the following order: Cs^+^ >NH_4_^+^ >K^+^ >Na^+^ >Li^+^, showing specific ion effects (Li et al., 2015; Hu et al., 2015b; Du et al., 2017). These sequences of ion effects are frequently observed in physicochemical phenomena. There are also cases close to the *lyotropic* or Hofmeister series (Van Olphen, 1977).

In addition, the semiquantitative analysis results showed that kaolinite was the main clay mineral component of the studied soils and reached 86.19% in red soil (Table 2). It is well known that kaolinite is a 1:1 phyllosilicate clay mineral, in which the basic layer consists of silicon-oxygen tetrahedra and aluminum-oxygen octahedra. The radius of the hexagonal structure of the silicon-oxygen tetrahedron was approximately 1.40 Å; therefore, NH_4_^+^ (1.43 Å) and K^+^ (1.33 Å) could be fixed into hexagonal holes to form a dense clay mineral structure, while Cs^+^ has a high selectivity and affinity with hexagonal holes, which would increase the stability of clay particles and aggregates, further increasing the soil shear strength (Dixon, 1989). However, the radii of Na^+^ (0.97 Å) and Li^+^ (0.68 Å) were much smaller than that of the hexagonal structure, meaning that they could not be fixed in the hexagonal hole. Thus, K^+^ NH_4_^+^ and Cs^+^ can increase the stability of soil, which may decrease the erosion intensity of Benggang and prevent the occurrence of Benggang.

### Specific ion effects on clay surface properties

The clay surface properties of the three different soil layers of the Benggang were quite different. The surface potential (*φ*_0_), specific surface areas (*S*) and surface charge number (*SCN*) were greater in the red soil than that in the sandy soil and detritus soil (Table 4). These results were in accordance with the report by Xiong, Chen & Zhang (1985). The obtained *S* values of the soils were reasonable, considering that kaolinite was the main clay mineral composition of the studied soils. For a given material, the *S* values was constant, and the *SCN* values was also constant for both permanently and variably charged particles under neutral pH solution conditions (Li et al., 2011). The *σ*_0_ and *E*_0_ of the three different soil layers showed opposite trends with *φ*_0_, *S* and *SCN* (Table 4). The results indicated that the *σ*_0_ and *E*_0_ values of red soil were lower than those of sandy soil and detritus soil. Strong soil electric fields can further affect soil particle interactions and reduce aggregate stability, implying that the sandy soil layer and detritus layer are more unstable under heavy rainfall than the red soil layer and are more susceptible to soil erosion.

The absolute zeta potential (*ζ*) and the shear plane thickness (*x*_*s*_) of the saturated clay particles in the studied soils was seriously effected by the concentration and the type of electrolyte solutions. The result showed that the absolute *ζ* and the *x*_*s*_ of clay particles decreased as the electrolyte concentration increased (Tables 5 and 6). This result was in accordance with that reported by Ding et al. (2015). The absolute *ζ* and the *x*_*s*_ of the saturated clay particles was obviously different in solutions with the same electrolyte concentration while saturated with Li^+^, Na^+^, K^+^, NH_4_^+^ and Cs^+^. The absolute *ζ* of clay particles of studied soils was followed the order of Cs^+^ >K^+^ >NH_4_^+^ >Na^+^ >Li^+^, while the *x*_*s*_ values of clay particles saturated with Li^+^ and Na^+^ were higher than those saturated with K^+^, NH_4_^+^and Cs^+^, indicating strong specific ion effects on zeta potential and shear plane thickness. As discussed above, the five cations had different hydrated radius, indicated they had different ability to shield the electric fields which arised from charged clay particles. Compared with Li^+^ and Na^+^, the smaller hydrated radius of K^+^, NH_4_^+^ and Cs^+^ had the better ability to shield the electric field strength, which maked them get more close to the surface of clay particles, then owned a better ability to compress the diffuse double layer and shear plane thickness (Li et al., 2015).

In summary, different monovalent cations showed strongly specific ion effects on the shear strength and clay surface properties of the studied soils. Compared with the clay particles of Li^+^- and Na^+^-saturated soils, the clay particles of K^+^-, NH_4_^+^- and Cs^+^-saturated soils had higher shear strengths and thinner shear plane thicknesses because K^+^, NH_4_^+^and Cs^+^ with a small ionic hydrated radius could shield more electric field strength, which significantly decreased the distance from the particle surface to the shear plane and suppressed the shear layer thickness. Then, deceased the electrostatic repulsion of the adjacent clay particles, thus increasing the aggregate stability and the shear strength, indicating that the shear strength has a close relationship with the clay surface properties. The results revealed that soils saturated with K^+^, NH_4_^+^ and Cs^+^ could increase the shear strength and the stability of the collapsing wall, thus might decrease the erosion intensity of Benggang. Therefore, to investigate the specific ion effects on the shear strength and clay surface propertie of collapsing walls is important for providing insight into the occurrence mechanisms and the treatment of Benggang erosion.

## Conclusions

In this study, the physicochemical and mineralogical properties of different granite residual soil of callapsing wall were investigated, meanwhile, different five monovalent cations (Li^+^, Na^+^, K^+^, NH_4_^+^ and Cs^+^) were used to systematically clarify the specific ion effects on the shear strength and clay surface properties of the different soil layers in the collapsing wall of a typical Benggang. The results show that red soil had physicochemical characteristics significantly superior to those of sandy soil and detritus soil, with higher CEC, SOM, clay content, plastic limit and liquid limit. The XRD analysis indicated that kaolinite was the major clay mineral component in the studied soils. Considering that clay particles were negatively charged, different monovalent cations showed intensified specific ion effects on the shear strength and clay surface properties of the studied soils after cation saturation treatments. The shear properties showed that the shear strength of K^+^-, NH_4_^+^- and Cs^+^-saturated soils has a better ability to resist shearing because of their greater cohesion (*c*), even though the internal friction angle (*φ*) had no significant difference from Li^+^- and Na^+^-saturated soils. Compared with Na^+^- and Li^+^, K^+^, NH_4_^+^ and Cs^+^with a small ionic hydration radius could shield more electric field strength surrounding the clay surface, which could decrease the shear plane thickness and cause a difference in shear strength, thus showing specific ion effects. Therefore, soil saturated with K^+^, NH_4_^+^ and Cs^+^ cations could increase the shear strength and collapsing wall stability and thus might decrease the erosion intensity of Benggang. In general, different monovalent cations can seriously affect the shear strength and clay surface properties of the granite residual soil layers of Banggang, and then plays an important role in the occurrence and development of Benggang erosion. This study considered different monovalent cations on the shear strength and clay surface properties; however, soil is a complex system that contains multivalence cations. Therefore, the effect of multivalent cations on the shear strength and clay surface properties also deserves further in-depth study.

##  Supplemental Information

10.7717/peerj.17796/supp-1 Supplemental Information 1 Raw data

## Additional Information and Declarations

### Data Availability

The following information was supplied regarding data availability: The raw measurements are available in the Supplementary Files.
